# Prognostic signature construction of energy metabolism-related genes in pancreatic cancer

**DOI:** 10.3389/fonc.2022.917897

**Published:** 2022-09-29

**Authors:** Hao Liu, Jianhua Zhang, Chaoguang Wei, Zhao Liu, Wei Zhou, Pan Yang, Yifu Gong, Yuxiang Zhao

**Affiliations:** ^1^ Key Laboratory of Marine Biotechnology of Zhejiang Province, School of Marine Sciences, Ningbo University, Ningbo, China; ^2^ College of Pharmaceutical Science, Zhejiang Chinese Medical University, Hangzhou, China; ^3^ United New Drug Research and Development Center, Hunan Biotrans Technology Co., LTD., Changsha, China; ^4^ Institute of Bioengineering, Biotrans Technology Co., LTD., Shanghai, China

**Keywords:** VAMP2, pancreatic cancer, energy metabolism, prognosis signature, consensus clustering

## Abstract

Pancreatic cancer is the 7th leading cause of cancer death worldwide, and its incidence and mortality rate have been on the rise in recent years in Western developed countries. The specificity of the disease and the lack of appropriate treatments have resulted in a 5-year overall survival rate of only 9%. In this study, we conducted a study based on the TCGA database and GEO database and analyzed using the energy metabolism gene set to establish a prognostic model with the least absolute shrinkage and selection operator to identify 7-genes prognostic signature, and the gene expression was verified by Real-time PCR. The model was validated using a risk score calculation, and the OS rates of the 7 genes were analyzed using one-way Cox regression. The prognostic relationship between vesicle-associated membrane protein 2 (VAMP2) and pancreatic cancer patients was analyzed by OS and progression-free survival, and the prognosis was found to be significantly worse in the high-expression group. A Nomogram showed that VAMP2 was an independent prognostic factor in pancreatic cancer. Gene set enrichment analysis showed that VAMP2 upregulation was enriched in pathways associated with immune response and that VAMP2 downregulation was enriched in metabolism-related pathways. The association of VAMP2 with immune cell infiltration was analyzed for the enrichment results, and VAMP2 was found to be positively associated with all 6 immune cells. The results of this study suggest that VAMP2 is an independent prognostic factor associated with energy metabolism in pancreatic cancer and may be involved in the immune response.

## Introduction

Pancreatic cancer ranks as the 14th most common cancer worldwide and is the 7th leading cause of cancer death (1). The incidence of pancreatic cancer is mainly concentrated in developed regions and is slightly higher in men than in women (2). According to Globolcan estimates, there will be more than 495,000 new cases and 466,000 deaths in 2020 (3), with a 5-year survival rate of only 9%. The low survival rate is mainly due to the fact that patients often present with symptoms at an advanced stage and to the lack of appropriate diagnostic tools and treatment measures (4). Patients with pancreatic cancer are usually classified as resectable, borderline resectable, locally advanced, or metastatic according to the degree of disease progression, with surgical resection being the only curative option (5). However, the vast majority of patients are diagnosed with inoperable advanced or metastatic disease, which to some extent reduces the prognostic survival time of pancreatic cancer patients (6). The development of pancreatic cancer is commonly associated with diabetes, as well as obesity, chronic pancreatitis, alcohol abuse, and genetic susceptibility (1).

The growth of cancer cells requires a large supply of energy, and to meet the demands of rapid growth, cancer cells reprogram their energy metabolism (7). This phenomenon is called “energy metabolic reprogramming” and is associated with the malignant biological behavior of pancreatic cancer (8). There are various ways to reprogram energy metabolism. Warburg suggested that cancer cells ferment glucose through glycolysis to obtain energy for growth (9). It is also possible to provide biomolecules for cell replication through the pentose phosphate and serine pathways, as well as using glutamine and lipids to promote their own proliferation (10). In the metastatic process of pancreatic cancer cells, metabolic reprogramming provides energy through aerobic glycolysis and oxidative phosphorylation, among other mechanisms (11). Cancers are heterogeneous diseases with complex and diverse metabolic patterns and the ability to improvise. Metabolic alterations contribute to the regulation of apoptosis and angiogenesis and confer a resistance phenotype (12). This resistance is reflected in pancreatic cancer in terms of drug resistance, which leads to poor treatment response (13). Metabolic reprogramming in pancreatic cancer is also associated with chemotherapy, radiotherapy, and immunotherapy, which can lead to poor prognosis (14).

The aim of this study was to identify prognostic features associated with energy metabolism in pancreatic cancer and to provide suggestions on the direction of pancreatic cancer treatment. We established 7-genes prognostic signature using the least absolute shrinkage and selection operator (LASSO) model and identified vesicle-associated membrane protein 2 (VAMP2) as a new energy metabolism-related prognostic biomarker for pancreatic cancer.

## Data and methods

### Data sources

Clinical information and the gene expression profiles of pancreatic cancer were obtained from the TCGA database (https://portal.gdc.cancer.gov/), containing 178 tumor samples and 4 normal samples. Since there were too few paraneoplastic samples in TCGA, 328 paraneoplastic samples were obtained from the GTEx database for subsequent analysis. Two energy metabolism-related gene sets containing 156 genes were downloaded from MSigDB (https://www.gsea-msigdb.org/gsea/msigdb/). GSE57495, GSE11838, GSE15932 and GSE62165 were obtained from the GEO database (https://www.ncbi.nlm.nih.gov/geo/), containing clinical information and genes expression profiles. Immunoscore data were obtained from the TIMER (http://timer.cistrome.org/).

### Consensus clustering

Consensus clustering was performed based on the TCGA-PAAD dataset and energy metabolism-related genes to compare the clinical information of different subgroups. Consistency analysis was performed using the R package ConsensusClusterPlus with a threshold of 2 clusters and 100 repetitions of 80% of the samples drawn. The clustering heat map was drawn using the R package pheatmap.

### Selection of prognosis-related energy metabolism genes

Genes associated with overall survival (OS) in pancreatic cancer patients were analyzed using a univariate COX model, and a forest plot was drawn using the R package forestplot to represent the top 20 most significant genes. The genes with significant prognosis were intersected with energy metabolism genes and visualized by a Venn plot.

### Construction of energy metabolism-related prognostic gene signature

Energy metabolism-related prognostic gene signature was constructed based on associations of gene expression levels with energy metabolism-related genes. The LASSO regression algorithm was used for gene signature selection, 10-fold cross-validation was applied, and a risk score model was constructed. Grouping was performed based on the best risk score cut-off value, and the expression and survival differences between the high- and low-risk groups were analyzed. The relationship between risk scores and clinical traits was analyzed by the univariate and multivariable Cox model. The TCGA dataset was used as the training set and GSE57495 was used as the validation set; P<0.05 was considered statistically significant.

### Gene expression validation and prognostic correlation analysis

The univariate and multivariable Cox model were used to analyze the relationship between clinical traits and OS in pancreatic cancer patients. R package ggplot2 was used to perform the box plot of VAMP2 expression under different clinical traits groups. The OS and progression-free survival (PFS) of prognostic traits were analyzed using the R package Survival, and the receiver operating characteristic (ROC) curve was plotted by timeROC. Human epidermoid tumor pancreatic ductal tumor cell line PANC-1, human pancreatic adenocarcinoma cell line BxPC-3 and human normal pancreatic ductal cell line hTERT-HPNE were used to verify the gene expression of the 7-genes prognostic signature by RT-PCR. The primer sequences were listed in Tab S1.

### Gene set enrichment analysis

GSEA was used to analyze functional differences between groups when considering gene expression (15). The KEGG database was used as the functional gene set for GSEA, with the cut-off threshold |NES|>1, NOM p-val<0.05, and FDR q-val<0.25.

### Immunocorrelation analysis

The relationship between the expression of VAMP2 and immune cells infiltration level was analyzed using Spearman, and immune cell correlation plots were performed with the R package ggstatsplot.

## Results

### Consensus cluster analysis of energy metabolism-related genes in pancreatic cancer

**Figure 1:**
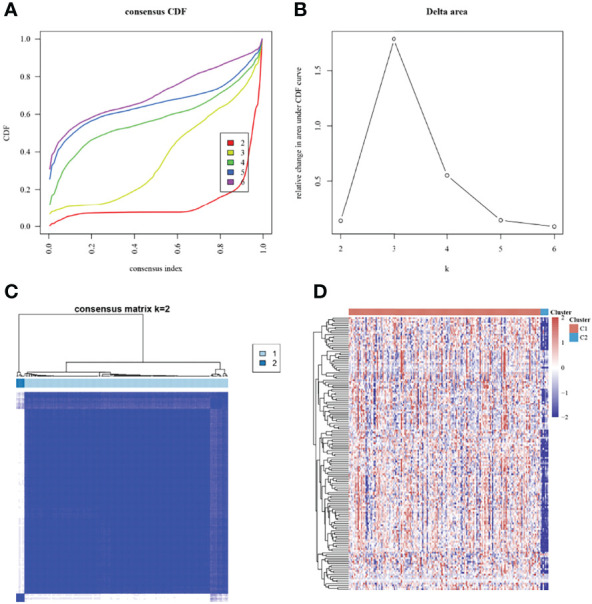
Consensus clustering analysis based on energy metabolism-related genes. **(A)**: Cumulative distribution function (CDF) curve; **(B)**: CDF delta area curve; **(C)**: Consensus matrix when K=2; **(D)**: Heat map of energy metabolism-related genes in different subgroups, with red indicating high expression, and blue indicating low expression.

The pancreatic cancer patients in TCGA were grouped based on the expression of energy metabolism-related genes and were divided into 2 subgroups (**Figures 1A–C**). The expression of energy metabolism-related genes in the 2 subgroups is shown in **Figure 1D**. A comparison of clinical information between the 2 subgroups revealed significant differences in performance in age, T stage, and tumor stage (**Table 1**).

**Table 1:** Comparison of clinical information.

Types	Charar	C1	C2	P value
Status	Alive	82	4	
	Dead	89	3	0.927
Age	Mean (SD)	64.5 (10.9)	71.3 (5.8)	
	Median [MIN, MAX]	65 [35,88]	72 [63,79]	0.02
Gender	FEMALE	77	3	
	MALE	94	4	1
Race	ASIAN	10	1	
	BLACK	6		
	WHITE	151	6	0.948
pT_stage	T1	7		
	T2	23	1	
	T3	137	5	
	T4	2	1	
	TX	1		0.037
pN_stage	N0	50		
	N1	112	7	
	N1b	4		
	NX	4		
pM_stage	M0	74	5	
	M1	5		
	MX	92	2	0.313
pTNM_stage	I	1		
	IA	5		
	IB	15		
	IIA	28		
	IIB	112	6	
	III	2	1	
	IV	5		0.414
Grade	G1	29	2	
	G2	94	1	
	G3	46	2	
	G4	1	1	
	GX	1	1	0
new_tumor_event_type	Metastasis	54	3	
	Metastasis:Recurrence	2		
	Primary	2		
	Recurrence	19	1	1
Smoking	Non-smoking	63	2	
	Smoking	75	4	0.861

### Construction of energy metabolism-related gene signature

**Figure 2:**
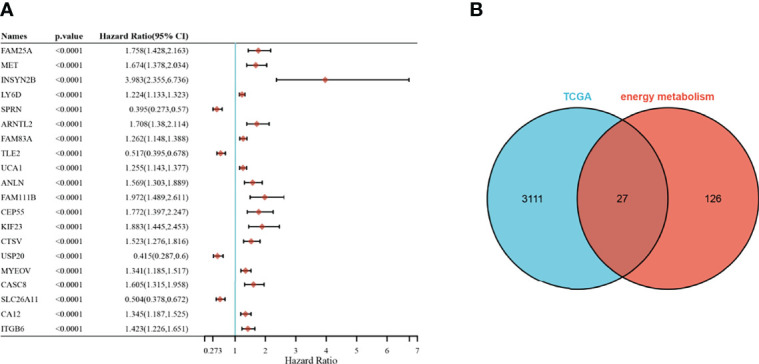
Selection of prognosis-related energy metabolism genes. **(A)**: Forest plot of the top 20 significant prognostic genes; **(B)**: Venn plot.

**Figure 3:**
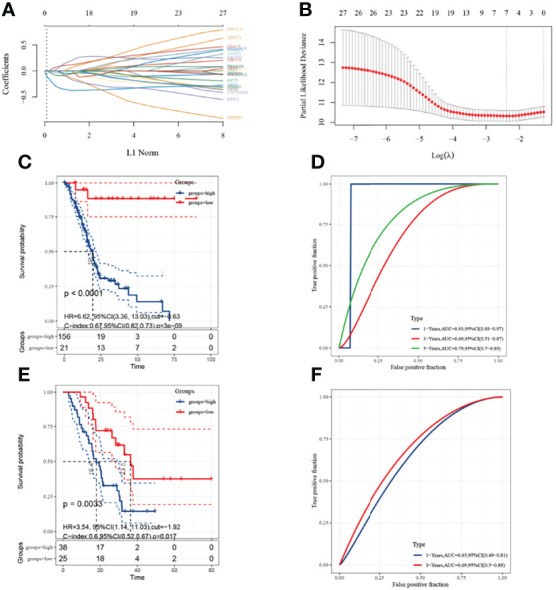
Construction of prognostic 7-gene signature with LASSO model. **(A)**: The coefficients of selected features are shown by lambda parameter. The horizontal axis represents the value of the independent variable lambda, and the vertical axis indicates the coefficient of the independent variable; **(B)**: Partial likelihood bias and log; **(C, D)**: KM survival curve and ROC curve based on the 7-gene signature in the training set; **(E, F)**: KM survival curve and ROC curve based on the 7-gene signature in the validation set. Due to the microarray samples, only the area under the curve (AUC) for 1 and 3 years is available in the ROC curves.

A univariate Cox analysis showed a total of 3138 genes associated with prognostic OS in pancreatic cancer (**Figure 2A**). These genes were intersected with energy metabolism-related genes to obtain 27 signature genes (**Figure 2B**). The 27 characteristic genes were used to construct the LASSO model, and 7 genes were obtained when the minimum characteristic coefficient (λ)=0.0918 (**Figures 3A, B**). The data risk scores in the set were calculated using the following risk score formula:


RiskScore=(−0.0133)∗ACACB+(0.0839)∗GNA15+(−0.3206)∗GNB3+(−0.0897)∗GNG7+(0.0601)∗IQGAP1+(−0.0145)∗STXBP1+(−0.036)∗VAMP2

The best cut-off value for the training set was −0.63. KM curves showed significant prognostic differences between the high- and low-risk groups in the training set (**Figure 3C**), and ROC analysis indicated AUCs of 0.93, 0.69, and 0.79 at 1, 3, and 5 years, respectively (**Figure 3D**). The model was validated using GSE57495 and showed significantly worse prognostic survival in the high-risk group when grouped using an optimal cut-off value of −1.92 (**Figure 3E**) and ROC showing AUCs of 0.65 and 0.69 at 1 and 3 years, respectively (**Figure 3F**).

### Validation of the 7 genes energy metabolism-related prognostic signature model

**Figure 4:**
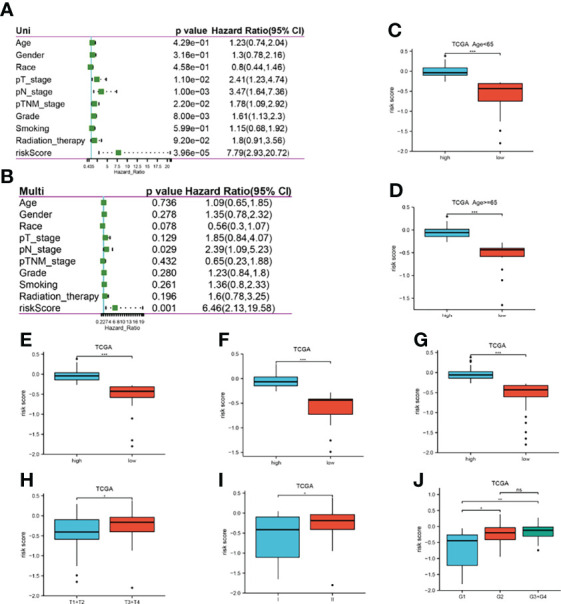
Correlation between risk scores and clinical traits in TCGA. **(A, B)**: P-value, HR, 95% CI for univariate and multifactorial Cox analysis of clinical traits and risk scores; **(C–J)**: Differences in expression of high and low risk scores in different clinical traits, respectively, age<65, age≥65, male, female, Caucasian, T stage, pTNM stage, and tumor grade. *P<0.05, **P<0.01, ***P<0.001, ns, no significant.

To validate the robustness of the LASSO model, P-values, HR values, and 95% CI of each clinical trait and risk score were analyzed by univariate and multifactorial Cox regression in the TCGA dataset, and the risk score was found to be an independent prognostic predictor for pancreatic cancer patients (**Figures 4A, B**). The high and low expression groups were classified according to the median risk score, and the differences in the high and low expression of risk score among different clinical traits were observed. The results showed that the high risk score group had high expression, which was associated with the degree of tumor differentiation (**Figures 4C–J**).

### Expression of 7 energy metabolism-related gene traits

**Figure 5:**
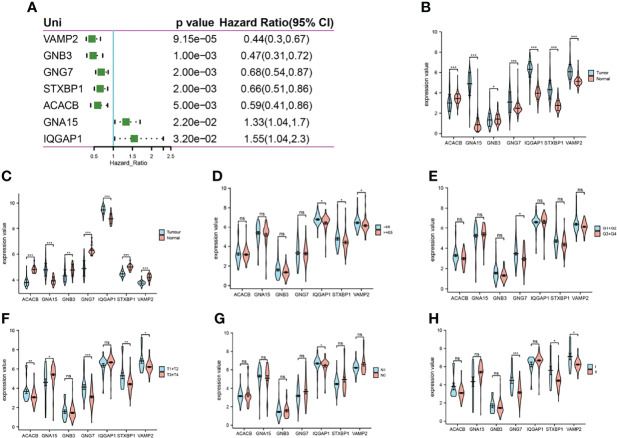
Expression of 7 genes. **(A)**: one-way Cox regression analysis of the prognosis of 7 energy metabolism-related genes in pancreatic cancer; **(B)**: expression differences between cancer and paracancer in TCGA; **(C)**: expression differences between cancer and paracancer in GSE62165; **(D–H)**: expression differences of genes in different age stages, grade stage, T stage, N stage, and pTNM stage in TCGA, respectively. *p<0.05, **p<0.01, ***p<0.001 compared with the control group. ns, no significant.

**Figure 6:**
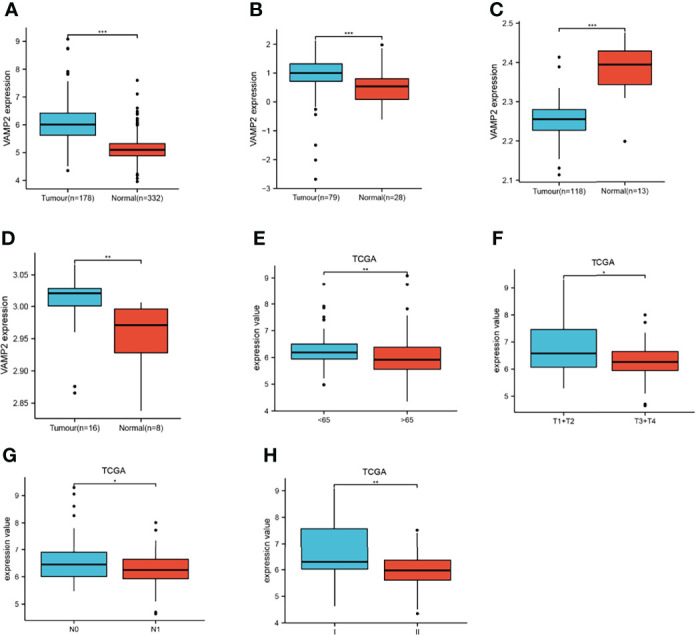
Expression of VAMP2. **(A)**: TCGA dataset as the training set, **(B–D)**: GSE11838, GSE62165, and GSE15932 as the validation set for analyzing the expression of VAMP2 in cancer versus paracancer and observing the expression differences between age, T stage, N stage, and pTNM stage **(E–H)**. *P<0.05, **P<0.01, ***P<0.001.

A one-way Cox regression analysis of the 7 energy metabolism-related gene features revealed that all were associated with prognosis in pancreatic cancer patients (**Figure 5A**). Subsequent analysis showed that all 7 trait genes were significantly differentially expressed in cancer versus paracancer (**Figures 5B, C**). However, the expression of the 7 energy-related signature genes differed across clinical traits, with VAMP2 expression being more prominent and significant for almost every clinical trait (**Figures 5D–H**). Expression analysis was performed for VAMP2, and differential expression was found in cancer versus paracancer and for each clinical trait (**Figure 6**). The expression levels of 7 genes in the cell lines were shown in **Figure S1**, which was consistent with the results in TCGA database.

### VAMP2 can be used as an independent predictor of pancreatic cancer

**Figure 7:**
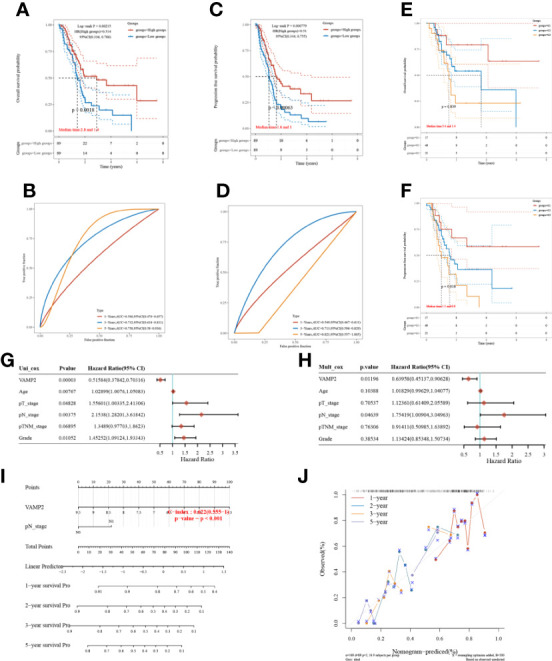
Prognosis of VAMP2. **(A, B)**: OS; **(C, D)**: PFSKM survival curves and ROC prediction model; **(E, F)**: KM curves for different tumor grades with OS and PFS, respectively; **(G, H)**: single-factor Cox regression analysis and multi-factor Cox regression analysis based on OS; **(I)**: column line graph; **(J)**: calibration curve.

The relationships between VAMP2 and both prognostic OS and PFS of pancreatic cancer patients were analyzed by grouping with median expression values. OS and PFS survival were significantly higher in the high expression group than in the low expression group (**Figures 7A, C**), and had better predictive ability (**Figures 7B, D**). **Figures 7E, F** shows that the higher the expression of VAMP2, the higher the tumor grade and the worse the prognosis for survival. Combined with the analysis of VAMP2 expression and other clinical traits, VAMP2 was found to be an independent prognostic factor for pancreatic cancer patients (**Figures 7G, H**). A nomogram dependent on OS-independent prognostic parameters in pancreatic cancer patients was also constructed (**Figures 7I, J**).

### Involvement of VAMP2 in immune response

**Figure 8:**
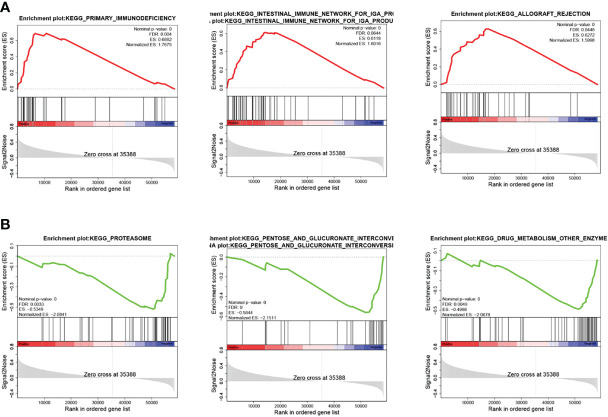
Pathway enrichment of VAMP2. **(A, B)**: KEGG pathway enrichment in high- and low-expression groups, respectively; hallmark pathway enrichment in high- and low-expression groups, respectively.

Based on the median expression value of VAMP2 for grouping, the functional pathways of the high- and low-expression groups were enriched using GSEA. The enrichment is shown in **Figure 8**. All pathways were ranked according to P-values. **Table 2** shows the top 10 significantly enriched pathways. The results show that when VAMP2 was highly expressed, it was mainly enriched in pathways related to immune response, and when VAMP2 was lowly expressed, it was mainly enriched in biometabolic pathways.

**Table 2:** KEGG pathway enrichment of top10.

	NAME	ES	NES	NOM p-val	FDR q-val
High_exp	primary immunodeficiency	0.688	1.767	0	0.004
	intestinal immune network for IGA production	0.612	1.602	0	0.064
	allograft rejection	0.627	1.600	0	0.045
	chemokine signaling pathway	0.552	1.569	0	0.043
	neuroactive ligand receptor interaction	0.540	1.553	0	0.046
	T cell receptor signaling pathway	0.552	1.540	0	0.048
	cell adhesion molecules cams	0.518	1.459	0	0.085
	cytokine cytokine receptor interaction	0.493	1.417	0	0.104
	calcium signaling pathway	0.499	1.413	0	0.103
	mapk signaling pathway	0.455	1.307	0	0.232
Low_exp	pentose and glucuronate interconversions	-0.584	-2.151	0	0.000
	proteasome	-0.535	-2.084	0	0.003
	drug metabolism other enzymes	-0.499	-2.068	0	0.005
	ascorbate and aldarate metabolism	-0.544	-2.021	0	0.004
	O glycan biosynthesis	-0.557	-1.989	0	0.005
	metabolism of xenobiotics by cytochrome p450	-0.440	-1.945	0	0.006
	retinol metabolism	-0.419	-1.863	0	0.011
	linoleic acid metabolism	-0.498	-1.826	0	0.012
	starch and sucrose metabolism	-0.419	-1.782	0	0.015
	pentose phosphate pathway	-0.484	-1.766	0	0.015

### VAMP2 is involved in immune infiltration in pancreatic cancer

**Figure 9:**
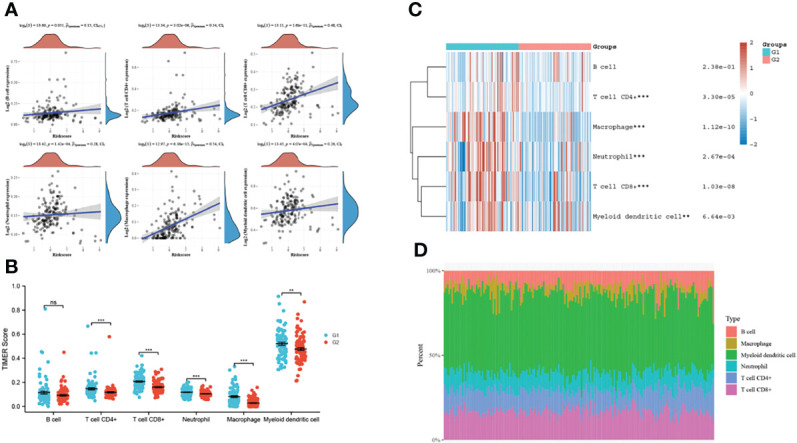
VAMP2 and immune correlation. **(A)**: relationship between VAMP2 and TIMER immune infiltrating cells; **(B)**: the relationship between VAMP2 and each immune cell score; **(C)**: heat map of immune cell scores; **(D)**: percentage abundance of tumor-infiltrating immune cells in each sample. **p<0.01, ***p<0.001 compared with the two groups. ns, no significant.

Since VAMP2, when highly expressed, was significantly enriched in immune-related pathways in pancreatic cancer, we performed an immune correlation analysis of VAMP2. As **Figure 9A** shows, VAMP2 in pancreatic cancer was positively correlated with all 6 types of immune infiltrating cells in TIMER. The strongest correlation was with macrophages. The samples were separated into high and low VAMP2 expression groups, and 5 immune cell scores were found to correlate with VAMP2 expression (**Figure 9B**). The heat map demonstrates the expression trends of different immune cell scores in different samples (**Figure 9C**), and **Figure 9D** shows the highest percentage abundance of myeloid dendritic cells.

## Discussion

The current modality that effectively prolongs the prognostic survival of patients with pancreatic cancer is surgical resection plus adjuvant chemotherapy (16). However, the vast majority of patients are not suitable for surgical resection and have a high recurrence rate, resulting in a median survival rate of only 2–2.5 years (17). As an aggressive cancer, pancreatic cancer has a high metabolism, which means that an adequate energy supply is required to meet the growth of cancer cells (18). Energy metabolic reprogramming, which maintains the energy balance during cancer cell growth, proliferation, and migration, is an emerging hallmark of cancer (19). Pancreatic cancer relies mainly on glutamine to maintain cell proliferation and survival. It also uses the glycolytic pathway to metabolize glucose, thereby producing ATP (20). It has been reported that energy metabolism can lead to the expression or silencing of specific oncogenes, resulting in abnormal cell proliferation, cycle arrest, and cellular senescence (21). LASSO prognostic modeling of energy metabolism-related genes revealed 7 prognostic features most associated with OS in pancreatic cancer patients. The risk scores calculated using the 7 prognostic features were determined as independent prognostic factors for pancreatic cancer patients and were validated using the GSE57495 data. These 7 prognostic features are GNB3, which has been shown to affect OS in pancreatic cancer (22); GNG7, which can be used as a therapeutic target in pancreatic cancer (23); IQGAP1, the overexpression of which promotes pancreatic cancer progression (24), and 4 other genes (ACACB GNA15, STXBP1, VAMP2) that have not yet been reported in pancreatic cancer.

VAMP2 is a protein-encoding gene that belongs to the family of synaptic vesicle proteins (VAMPs) responsible for intracellular transport and extracellular secretion of vesicles (25). VAMP2 is an abundant synaptic vesicle protein that is closely associated with cancer cell adhesion, survival, and migration. Reduced expression of VAMP2 can lead to useless protein degradation and abnormal patterns of unwanted protein degradation (26). Recently, it has been shown that VAMP2 is significantly expressed in bladder cancer and increases in a stage-dependent manner according to tumor stage (27). VAMP2 acts as a downstream target and plays a pro-tumorigenic role in liver cancer (25). It also affects ovarian cancer prognosis and tumor progression (28). In addition, VAMP2 can act as a fusion gene and play an oncogenic role in non-small cell lung adenocarcinoma (29). In the present study, VAMP2 was screened as an energy metabolism-related feature, and GSEA showed that down-regulated VAMP2 was mainly enriched in glucose metabolism-related pathways, such as the pentose and glucuronide interconversion pathway and the pentose phosphate pathway (PPP). The PPP is a major regulator of cellular redox homeostasis and biosynthesis and is an important component of glucose metabolism (30). It also supports the glycolytic process in cancer cells, helping to meet their anabolic demands and counteract oxidative stress (31). PPP flux plays a role in promoting cancer cell survival and proliferation and is associated with the progression of hepatocellular carcinoma, lung cancer, and breast cancer (32). This suggests that VAMP2 downregulation may also affect pancreatic cancer progression by regulating the PPP.

GSEA showed that upregulated VAMP2 was mainly enriched in immune response-related pathways, such as chemokine signaling pathways and cytokine–cytokine receptor interactions. Based on this result, we analyzed VAMP2 along with immune cells and immune scores and found that VAMP2 was positively correlated with all 6 immune cells in TIMER and showed differential expression in multiple immune cell scores. The tumor microenvironment (TME) has multiple components, among which immune cells and cytokines are important and inextricably linked to tumor progression (33). Cytokines are molecular messengers of innate and adaptive immunity that allow immune cells to communicate in a paracrine or autocrine manner (34). Cytokines inhibit tumor cell growth by suppressing proliferation, promoting apoptosis, or stimulating the toxic activity of immune cells against tumor cells (35). Pro-inflammatory cytokines can promote cancer immunotherapy, acting at each stage of the cancer immune cycle (36). It has been shown that VAMP2 is the main type of VAMP that is functionally involved in antibody secretion (37). VAMP2 is a key protein in the SNARE complex that mediates the release of neurotransmitters from synaptic vesicles by neurons (38). Complexes of VAMP2 can lead to inflammatory pain in the dorsal horn of the spinal cord, which implies that VAMP2 may have an inflammatory role (39). The GSEA results suggest that it may be possible to mediate the immune response of immune cells by up-regulating the expression of VAMP2 to produce a therapeutic effect on pancreatic cancer.

In this study, we used bioinformatics to identify prognostic genes associated with energy metabolism in pancreatic cancer. We then constructed prognostic models to identify signature genes and validated them using an external validation set. The results of an expression analysis of different clinical traits led us to focus on VAMP2, and a prognostic analysis confirmed that VAMP2 is an independent prognostic factor in pancreatic cancer. GSEA demonstrated the VAMP2-enriched KEGG pathway and preliminary analysis of the association between VAMP2 and immune response. In conclusion, we believe that this study will provide new knowledge for the precise treatment of pancreatic cancer and provide a new strategy for predicting the survival of pancreatic cancer patients based on the expression of energy metabolism-related genes.

## Data availability statement

The datasets presented in this study can be found in online repositories. The names of the repository/repositories and accession number(s) can be found in the article/**Supplementary Material**.

## Author contributions

YZ and YG designed this study. JF, JZ, CW and ZL performed the analysis. HL and YZ wrote the manuscript. WZ provided constructive suggestions for the structure of the manuscript and the visualization of the analysis. All authors contributed to the article and approved the submitted version.

## Funding

This study was supported by Opening Project of Zhejiang Provincial Preponderant and Characteristic Subject of Key University (Traditional Chinese Pharmacology), Zhejiang Chinese Medical University (No. ZYAOXYB2019002) and the K.C.Wong Magna Fund in Ningbo University.

## Conflict of interest

HL, YZ, JZ, CW, ZL and PY are employed by Biotrans Technology Co., LTD.

The remaining authors declare that the research was conducted in the absence of any commercial or financial relationships that could be construed as a potential conflict of interest.

## Publisher’s note

All claims expressed in this article are solely those of the authors and do not necessarily represent those of their affiliated organizations, or those of the publisher, the editors and the reviewers. Any product that may be evaluated in this article, or claim that may be made by its manufacturer, is not guaranteed or endorsed by the publisher.
