# Supplementary material for: Prognostic signature construction of energy metabolism-related genes in pancreatic cancer
Source: Front Oncol. 2022 Sep 29;12:917897. doi: 10.3389/fonc.2022.917897 (PMC9559226; doi:10.3389/fonc.2022.917897)
Supplement: Supplementary file 1 [file DataSheet_1.docx]

Table S1 Primer sequences information.

| Genes | Primer sequences |
| --- | --- |
| ACACB | Forward: 5’-CAAGCCGATCACCAAGAGTAAA-3’ |
|  | Reverse: 5’-CCCTGAGTTATCAGAGGCTGG-3’ |
| GNA15 | Forward: 5’-CCAGGACCCCTATAAAGTGACC-3’ |
|  | Reverse: 5’-TGAATCGAGCAGGTGGAAT-3’ |
| GNB3 | Forward: 5’-CGGACGTTAAGGGGACACC-3’ |
|  | Reverse: 5’-CGAGGCACTTACCAGCAGC-3’ |
| GNG7 | Forward: 5’-ATGTCAGCCACTAACAACATAGC-3’ |
|  | Reverse: 5’-AGACCTTGATGCGCTCAATCC-3’ |
| IQGAP1 | Forward: 5’-AGAACGTGGCTTATGAGTACCT-3’ |
|  | Reverse: 5’-CCAGTCGCCTTGTATCTGGT-3’ |
| STXBP1 | Forward: 5’-AAAGCTGTTGTCGGAGAGAAG-3’ |
|  | Reverse: 5’-CACAATCGTTATGCCCTCGG-3’ |
| VAMP2 | Forward: 5’-CTCAAGCGCAAATACTGGTGG-3’ |
|  | Reverse: 5’-TGATGGCGCAAATCACTCCC-3’ |
| GAPDH | Forward: 5’-TGTGGGCATCAATGGATTTGG-3’ |
|  | Reverse: 5’-TGTGGGCATCAATGGATTTGG-3’ |
