# Supplementary figures and images for: Prognostic signature construction of energy metabolism-related genes in pancreatic cancer
Source: Front Oncol. 2022 Sep 29;12:917897. doi: 10.3389/fonc.2022.917897 (PMC9559226; doi:10.3389/fonc.2022.917897)

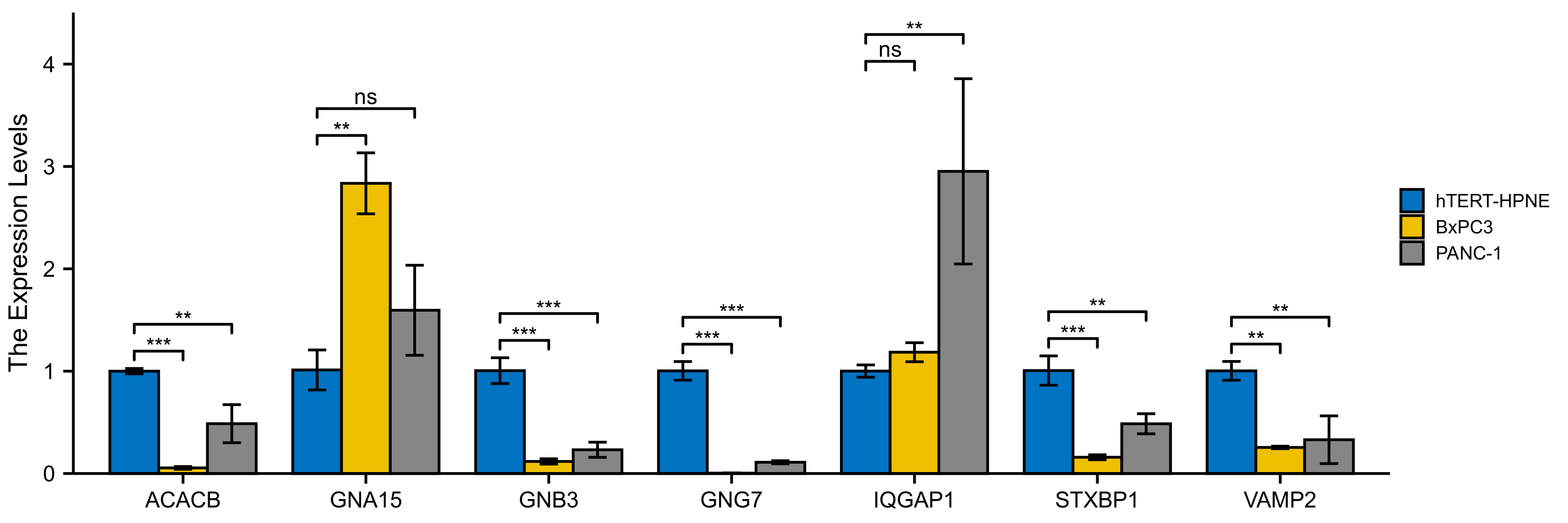

Supplement: Supplementary file 2 [file Image_1.tiff]
